# Oral health status in children with autism spectrum disorder in Almaty, Kazakhstan

**DOI:** 10.3389/fdmed.2026.1928744

**Published:** 2026-09-16

**Authors:** Zh. Yerkibayeva, G. Yermukhanova, K. Saduakasova, S. Yessembayeva, Y. Menchisheva, K. Rakhimov, Zh. Abu

**Affiliations:** 1 Asfendiyarov Kazakh National Medical University, Almaty, Kazakhstan; 2 Al-Farabi Kazakh National University, Almaty, Kazakhstan; 3 King’s College London, Franklin Wilkins Building, London, United Kingdom

**Keywords:** autism spectrum disorder, children, dental behavior management, dental care, Kazakhstan, oral health

## Abstract

**Purpose:**

This study aimed to characterize oral health status, oral hygiene practices, and caregiver-reported dental care experiences among children with autism spectrum disorder (ASD) in Almaty, Kazakhstan, and to describe caregiver awareness and practices related to oral care.

**Methods:**

This descriptive cross-sectional pilot study included 53 children aged 3-18 years with a confirmed ASD diagnosis recruited from the “Autism Pobedim” rehabilitation center in Almaty. Clinical examinations assessed caries using the DMFT/dmft and CAST indices and oral hygiene using the OHI-S index. A study-specific 36-item caregiver questionnaire collected information on home oral hygiene, dental care experiences, and selected developmental characteristics.

**Results:**

Only 24.5% of children brushed their teeth twice daily, and most required parental assistance. Overall, 30.2% had never visited a dentist, while 58.5% of caregivers reported major difficulties during dental visits. Reported management approaches in the sample included physical restraint, sedation, and general anesthesia. Overall, 39.6% of children had received completed dental treatment. In addition, 71.7% consumed sweets between meals and only 17.0% rinsed their mouths after eating. Although 75.5% of caregivers reported checking toothpaste composition, professional guidance on oral hygiene was limited.

**Conclusion:**

In this single-center sample, children with ASD had a substantial burden of dental caries, suboptimal home oral hygiene, limited independent oral care, and caregiver-reported challenges during dental treatment. These exploratory findings support larger multicenter studies and further evaluation of tailored preventive, caregiver-education, and behavior-support strategies for children with ASD in Kazakhstan.

## Introduction

The health and well-being of children and adolescents are closely linked to oral health, which influences eating, communication, social interaction, and quality of life (1–3). Children with developmental conditions such as autism spectrum disorder (ASD) may require adapted preventive and dental care approaches because of differences in communication, sensory processing, behavior, and daily self-care.

ASD is a neurodevelopmental condition characterized by persistent differences in social communication and interaction together with restricted or repetitive patterns of behavior or interests. Clinical presentation and support needs vary substantially among individuals. The World Health Organization has estimated that approximately one in 100 children is affected by ASD (4).

ASD is not associated with a single specific oral pathology; however, sensory sensitivities, communication difficulties, resistance to changes in routine, motor coordination limitations, and selective eating may complicate home oral hygiene and dental attendance (5–14). Studies from several countries have reported poor oral hygiene, considerable caries experience, frequent need for caregiver assistance with tooth brushing, and difficulties with cooperation during dental care among children with ASD, although comparisons with neurotypical children have been heterogeneous (2, 3, 5, 15–17).

Evidence from Central Asia remains limited, and there are few clinical data describing the oral health status and dental care experiences of children with ASD in Kazakhstan. Context-specific information is needed to characterize oral health needs and to guide the design of future preventive and service-delivery research.

Accordingly, this study aimed to describe the clinical oral health status and oral hygiene of children with ASD in Almaty using standardized dental indices and to characterize caregiver-reported home oral hygiene practices, dental care experiences, and perceived barriers to care. The study was designed as an exploratory descriptive investigation and did not aim to establish causal relationships or to evaluate the effect of ASD severity on oral health or treatment outcomes.

To our knowledge, this is among the first studies to report clinical oral health data for children with ASD in Kazakhstan, thereby providing preliminary evidence from an underrepresented geographical setting.

## Methods

### Study design and participants

This descriptive cross-sectional pilot study was conducted among children attending the “Autism Pobedim” rehabilitation center in Almaty, Kazakhstan. Children aged 3-18 years with a verified diagnosis of ASD in accordance with the Diagnostic and Statistical Manual of Mental Disorders, Fifth Edition (DSM-5), were eligible for inclusion. Children with systemic comorbidities that could independently affect oral health (e.g., cerebral palsy, syndromic disorders, severe epilepsy) were excluded to reduce potential confounding.

ASD severity was not assessed or stratified because standardized instruments such as CARS-2 or ADOS were not routinely available in the participating institutions at the time of data collection. Therefore, no analyses concerning the relationship between ASD severity and oral health or dental treatment outcomes were performed.

Given the exploratory pilot nature of the study, a formal *a priori* power calculation was not performed. Age-related variation in oral hygiene was described by age-based dentition groups: primary dentition (3-5 years), mixed dentition (6-11 years), and permanent dentition (12-18 years).

The study was approved by the Local Ethics Committee of S.D. Asfendiyarov Kazakh National Medical University [Approval No. 8(114), dated 30.06.2021]. All procedures involving human participants were conducted in accordance with institutional and national ethical standards and the Declaration of Helsinki. Written informed consent was obtained from the parents or legal guardians of all participating children before inclusion.

### Clinical measures

Dental examinations were performed at the rehabilitation center in the presence of a parent or caregiver. Disposable colored plastic mirrors and probes were used. Oral status was assessed using the Decayed, Missing and Filled Teeth indices for primary and permanent dentitions (dmft/DMFT) and the Caries Assessment Spectrum and Treatment (CAST) index. Oral hygiene was evaluated using the Simplified Oral Hygiene Index (OHI-S; Greene and Vermillion), and dental plaque was additionally visualized using the Qscan Plus device (AIOBIO, South Korea).

CAST was used to record the spectrum of caries from sound or restored teeth through enamel and dentinal lesions to advanced disease and tooth loss due to caries. Scoring followed the published CAST methodology (18); detailed diagnostic codes are therefore not repeated here.

### Caregiver questionnaire

Caregivers completed a structured 36-item questionnaire in Google Forms. The questionnaire was developed specifically for this study and had not been previously published. It collected sociodemographic information and caregiver-reported data on tooth-brushing frequency and assistance, toothpaste and toothbrush practices, sources of oral-health information, dental attendance and treatment experiences, difficulties during dental visits, dietary habits, and selected developmental characteristics, including age at ASD diagnosis (Supplementary File S1).

The questionnaire was intended as a descriptive data-collection instrument rather than a psychometric scale. Formal assessment of construct validity, test-retest reliability, or internal consistency was not performed. Accordingly, questionnaire-derived findings were interpreted as exploratory caregiver-reported data.

### Statistical analysis

Data were processed using Microsoft Excel and IBM SPSS Statistics version 26.0. Descriptive statistics were used to summarize demographic, clinical, and questionnaire data. Categorical variables are presented as absolute frequencies (n) and percentages (%), while quantitative variables are summarized using means, standard deviations, medians, and ranges where appropriate.

OHI-S values were summarized by the three age-based dentition groups. Because of the small sample and sparse cell counts, inferential analyses were limited. For one exploratory comparison, tooth-brushing frequency was dichotomized as daily (once or twice daily) vs. less than daily, and its association with history of dental attendance (visited a dentist vs. never visited) was assessed using Fisher's exact test. A two-sided *p*-value < 0.05 was considered statistically significant. All other questionnaire analyses were descriptive.

Missing or incomplete responses were excluded from the relevant item-level analysis while the overall sample size was retained for reporting.

## Results

This study included 53 children diagnosed with ASD, aged 3–18 years (mean age 8.26 ± 3.68 years), who attended a rehabilitation center in Almaty, Kazakhstan. Of the participants, 84.9% (*n* = 45) were male and 15.1% (*n* = 8) were female.

The mean number of decayed teeth was 3.85 ± 1.60 (range 1-8), the mean number of missing teeth was 0.38 ± 0.63 (range 0-2), and the mean number of filled teeth was 0.91 ± 1.20 (range 0–4). The mean DMFT/dmft index was 5.13 ± 2.05, with a median of 5 and a range of 2-10.

CAST assessment showed lesions across several stages. Mean values were 1.53 ± 0.72 for CAST code 3, 1.55 ± 1.03 for CAST code 4, and 0.68 ± 0.78 for CAST code 5; other CAST categories were less frequent.

All three age-based dentition groups had poor or very poor OHI-S values: 4.20 ± 0.15 in the primary dentition group, 3.95 ± 0.13 in the mixed dentition group, and 4.15 ± 0.14 in the permanent dentition group (Table 1). Values were comparably high across groups, with no monotonic age-related pattern apparent in these descriptive data. No inferential comparisons between dentition groups were performed.

**Table 1:** OHI-S by age-based dentition group.

Parameter	Primary dentition (3–5 years)	Mixed dentition (6–11 years)	Permanent dentition (12–18 years)
Mean OHI-S	4.20	3.95	4.15
Standard deviation	±0.15	±0.13	±0.14
Hygiene level	Poor	Poor	Very poor

Mean index values, standard deviations, and hygiene-level classifications across primary, mixed, and permanent dentition groups in children with ASD.

Caregiver responses indicated inconsistent home oral hygiene practices. Only 24.5% (*n* = 13) of children brushed twice daily, 41.5% (*n* = 22) brushed once daily, 22.6% (*n* = 12) brushed several times per week or occasionally, and 11.3% (*n* = 6) rarely or never brushed. Regarding assistance, 56.6% (*n* = 30) brushed with parental assistance, 28.3% (*n* = 15) were brushed solely by parents, only 3.8% (*n* = 2) brushed independently, and 11.3% (*n* = 6) did not brush their teeth.

Most children (64.2%, *n* = 34) used children's toothpaste, while 24.5% (*n* = 13) used adult toothpaste. Natural-based products were used by 3.8% (*n* = 2), both children's and adult toothpaste by 3.8% (*n* = 2), and no toothpaste by 3.8% (*n* = 2). A total of 75.5% (*n* = 40) of caregivers reported checking toothpaste composition. Toothbrushes were replaced every three months by 52.8% (*n* = 28), every six months by 37.7% (*n* = 20), annually by 3.8% (*n* = 2), and monthly by 5.7% (*n* = 3). Only one child (1.9%) used an electric toothbrush.

Information about oral hygiene was obtained primarily from the internet or social networks (49.1%, *n* = 26), followed by dental visits (22.6%, *n* = 12). However, 15.1% (*n* = 8) of caregivers reported having no information on how to properly brush their child's teeth. Overall, 69.8% (*n* = 37) of children had been examined or treated by a dentist, whereas 30.2% (*n* = 16) had never visited one. The most common reported reasons for dental attendance were caries or plaque (37.7%, *n* = 20) and acute dental pain (24.5%, *n* = 13); only 7.5% (*n* = 4) reported routine check-ups.

Regarding the timing of the most recent dental visit, 22.6% (*n* = 12) had last visited more than one year earlier and 20.8% (*n* = 11) approximately six months earlier. Major difficulties during dental visits were reported by 58.5% (*n* = 31) of caregivers, including fear and restlessness. Overall, 39.6% (*n* = 21) of children had received completed dental treatment. Reported behavior-management or treatment approaches included physical restraint (*n* = 7), general anesthesia (*n* = 5), and sedation (*n* = 6). In addition, 20.8% (*n* = 11) had previously undergone a dental procedure under general anesthesia.

Dental treatment had been provided in state clinics for 41.5% (*n* = 22) of the sample and in private clinics for 28.3% (*n* = 15); 30.2% (*n* = 16) had not received treatment. Caregivers reported recognizing possible dental problems through behaviors or signs such as putting fingers in the mouth or touching the cheeks (28.3%, *n* = 15), visible cavities (15.1%, *n* = 8), and complaints of pain (15.1%, *n* = 8). A smaller number reported swelling or eating difficulties, while 9.4% (*n* = 5) reported no dental problems.

Only 17.0% (*n* = 9) of children rinsed their mouths after meals, and 71.7% (*n* = 38) consumed sweets between meals; 24.5% (*n* = 13) consumed sweets daily or more often. Oral care was initiated at age three in 28.3% (*n* = 15), at age two in 17.0% (*n* = 9), and at age one in 15.1% (*n* = 8), while 9.4% (*n* = 5) had never started routine oral care.

When asked what could improve dental care, caregivers most frequently identified treatment under anesthesia or sedation (18.9%, *n* = 10), a more individualized approach (13.2%, *n* = 7), improved access or financial support (11.3%, *n* = 6), and better training for dental professionals (7.5%, *n* = 4). These responses represent caregiver perceptions and were not used as measures of healthcare-system performance.

Figure 1 summarizes selected oral hygiene and dental care utilization indicators. Figure 2 presents the distribution of dental attendance according to tooth-brushing frequency. In the exploratory dichotomized analysis, 25 of 35 children who brushed daily had visited a dentist compared with 12 of 18 children who brushed less than daily. Fisher's exact test did not show a statistically significant association between daily tooth brushing and history of dental attendance (*p* = 0.759).

**Figure 1:**
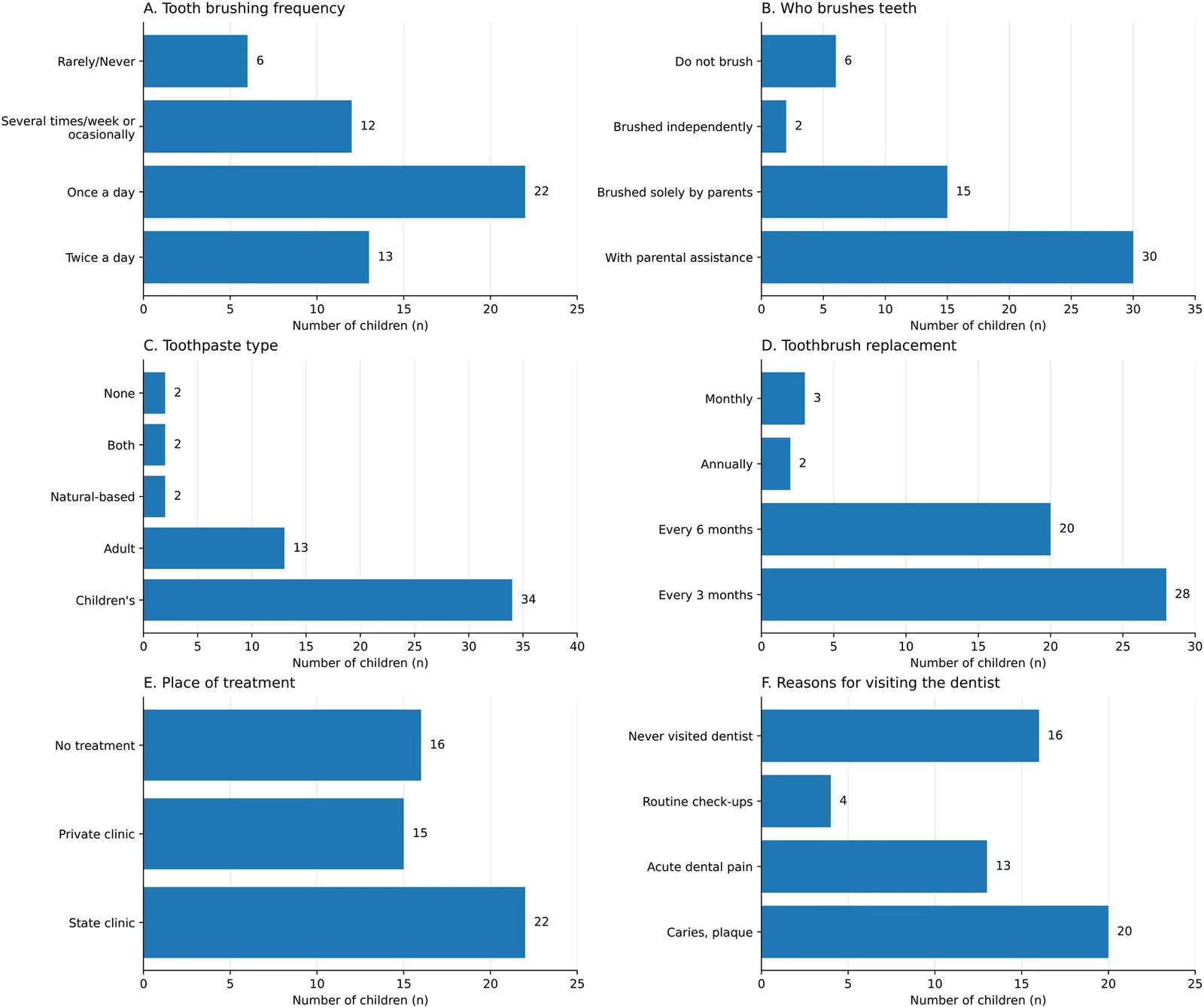
Oral hygiene practices and dental care utilization among children with ASD (*n* = 53): **(A)** tooth brushing frequency; **(B)** who brushes teeth; **(C)** toothpaste type; **(D)** toothbrush replacement; **(E)** place of treatment; and **(F)** reasons for visiting the dentist. Bars represent the number of children (*n*).

**Figure 2:**
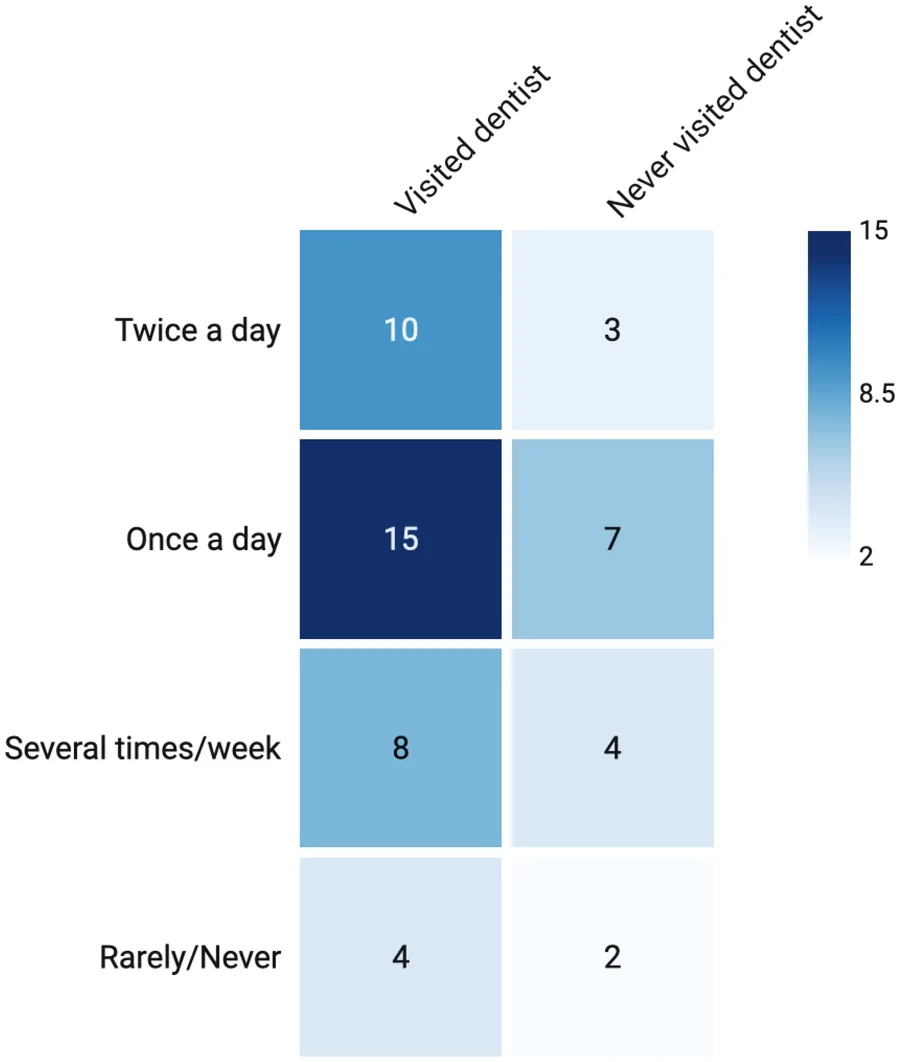
Distribution of dental attendance according to tooth-brushing frequency among children with ASD (*n* = 53).

## Discussion

This study provides preliminary clinical and caregiver-reported data on oral health among children with ASD in Almaty, Kazakhstan. The principal findings were a substantial caries burden, poor or very poor OHI-S values across all age-based dentition groups, low prevalence of twice-daily tooth brushing and independent oral care, frequent intermeal consumption of sweets, limited routine dental attendance, and caregiver-reported difficulties during dental visits. Because the study was descriptive and cross-sectional, these findings should be interpreted as co-occurring characteristics of this sample rather than evidence of causal relationships.

The mean DMFT/dmft value of 5.13 ± 2.05 was higher than values reported by Bagattoni et al. in an Italian ASD cohort (3.00 ± 1.2 in primary dentition and 2.3 ± 1.8 in permanent dentition) (3) and slightly higher than the mean value of 4.75 ± 3.66 reported by Oda et al. in Turkey (19). In the present sample, OHI-S values exceeded 3.9 in all dentition groups, and CAST assessment showed both superficial and deeper lesions. These observations are broadly consistent with previous reviews and observational studies describing poor oral hygiene and a considerable burden of dental disease among children with ASD (16, 17). Differences among studies may reflect variation in age, diagnostic criteria, oral-health measures, sampling, healthcare access, and local preventive practices.

Home oral hygiene was strongly dependent on caregiver involvement in this cohort: most children required assistance or complete parental brushing, whereas only two children brushed independently. Similar dependence on caregiver assistance has been reported in other ASD populations, including the study by Suhaib et al. (20). At the same time, nearly half of caregivers in our sample obtained oral-health information from the internet or social networks, and 15.1% reported receiving no guidance on how to brush their child's teeth. These findings identify caregiver education and practical home-based support as relevant areas for future intervention research; however, the present study did not test whether caregiver knowledge or brushing assistance caused differences in clinical oral-health outcomes.

Behavioral and sensory challenges during dental care were prominent in caregiver reports. Major difficulties during dental visits were reported for 58.5% of the sample, and caregivers described use of physical restraint, sedation, and general anesthesia in some children. Previous research has similarly identified cooperation, sensory responsiveness, and communication as important considerations in dental care for children with ASD (5, 15, 21). Importantly, recent studies have examined structured alternatives intended to improve treatment acceptance. Corridore et al. evaluated an educational sensory-based approach in children with ASD and reported improvement in cooperation across program phases, particularly among initially uncooperative children (22). Di Giorgio et al. also reported improved Frankl cooperation scores during orthodontic care delivered within a specialized supportive framework (23). In addition, the systematic review by Corridore et al. summarized the variability of oral disease burden and the treatment approaches used in pediatric dental care for children with ASD, including the role of general anesthesia in selected cases (24). These studies support further evaluation of individualized sensory adaptation, visual preparation, gradual desensitization, structured visits, and caregiver participation; these approaches were not tested in the current study.

Dietary and dental-attendance findings also warrant cautious interpretation. More than 70% of children consumed sweets between meals and only 17% rinsed after meals. These behaviors are clinically relevant to caries prevention, but the present design cannot determine their causal contribution to the observed caries burden. Similarly, 30.2% of children had never visited a dentist and only 7.5% of the total sample attended for routine check-ups. The exploratory analysis did not identify a statistically significant association between daily tooth brushing and history of dental attendance (*p* = 0.759), suggesting that these aspects of care should be considered separately rather than interpreted as directly linked within this dataset.

The broad age range is another important consideration. OHI-S was described by age-based dentition group, and all three groups had high mean values; however, the data did not show a monotonic age-related trend, and the sample was too small for detailed age-stratified analyses of caries, dietary behavior, dental attendance, or cooperation. Likewise, ASD severity was not measured using a standardized instrument. Although previous studies have explored developmental profiles and cooperation with oral-health procedures (21, 25–35), the present dataset cannot determine whether ASD severity influenced oral-health status, behavior during dental care, or treatment completion.

### Clinical implications and future directions

The caregiver-reported priorities in this study—including individualized care, improved access or financial support, and better professional training—may inform hypotheses for future service-development research. They should not, however, be interpreted as direct evidence that specific healthcare policies or service models will improve outcomes.

Future multicenter studies in Kazakhstan should recruit larger samples, include age- and, where feasible, ASD-severity stratification, use validated caregiver-reported instruments, and include comparison groups when the research question requires them. Prospective studies could evaluate sensory-adapted environments, visual supports, preparatory or “acclimatization” visits, caregiver training, and other structured behavioral approaches using clinically meaningful outcomes such as cooperation, treatment completion, need for pharmacological behavior management, and oral-health indices. Such studies would provide a stronger evidence base for locally appropriate clinical pathways and public-health planning.

### Limitations

This study has several limitations. First, the sample was small and recruited from a single rehabilitation center in Almaty; therefore, the findings may not be representative of all children with ASD in Almaty or Kazakhstan. The pilot design and absence of an *a priori* power calculation also limited the scope of inferential analyses.

Second, participants ranged from 3 to 18 years, spanning primary, mixed, and permanent dentitions and markedly different developmental stages. Only OHI-S was stratified by age-based dentition group, while most questionnaire and clinical findings were analyzed for the pooled sample. More detailed age-stratified analyses were not feasible because of small subgroup sizes.

Third, ASD severity was not assessed using standardized tools such as CARS-2 or ADOS; consequently, the relationship between ASD severity and oral health, cooperation, access to care, or treatment outcomes could not be evaluated. Fourth, the caregiver questionnaire was developed specifically for this study and did not undergo formal psychometric validation or reliability testing. Caregiver-reported findings may therefore be affected by recall, interpretation, or social-desirability bias.

Finally, the cross-sectional design does not permit causal inference, and the absence of a neurotypical or other disability comparison group limits comparative interpretation. Future studies should address these limitations using larger multicenter samples, validated instruments, prespecified age and ASD-severity strata, and longitudinal or comparative designs where appropriate.

## Conclusion

In this single-center Almaty sample, children with ASD showed a substantial burden of dental caries, poor oral hygiene across age-based dentition groups, limited independent tooth brushing, frequent intermeal sweet consumption, and caregiver-reported difficulties during dental care. These findings describe unmet oral-health needs but do not establish causal relationships or the influence of ASD severity.

The study provides preliminary data from an underrepresented setting and supports further multicenter research in Kazakhstan. Future work should evaluate age- and severity-related differences and test tailored caregiver-education, preventive, sensory-adapted, and behavioral-support strategies using validated outcome measures.

## Data Availability

The original contributions presented in the study are included in the article/Supplementary Material, further inquiries can be directed to the corresponding author.
