# Supplementary material for: Oral health status in children with autism spectrum disorder in Almaty, Kazakhstan
Source: Front Dent Med. 2026 Sep 16;7:1928744. doi: 10.3389/fdmed.2026.1928744 (PMC13623670; doi:10.3389/fdmed.2026.1928744)
Supplement: Supplementary file 1 [file Datasheet1.docx]

**SURVEY "ANALYSIS OF HEALTH OF THE ORAL CAVITY**

**OF CHILDREN WITH AUTISM"**

**DEAR SURVEY PARTICIPANTS!**

This questionnaire is conducted to study the dental health of children with autism spectrum disorder in different regions of the Republic of Kazakhstan.

Please be sure to answer questions more in detailed. This questionnaire is anonymous and will be considered in the general data set.

Data will help specialists working with children with autism.

1. Gender of the child 1 - boy 2 - girl

2. Date of birth of the child (dd.mm.yy) __________

3. At what age did you start oral care (brushing child’s teeth)?_______

4. Who performs the process of brushing teeth?

• Parents-

• Child with parental involvement-

• Child on his/her own-

• Child does not brush teeth -

• Other _____

5. How often does the child brush his/her teeth?

• once a day -

• twice a day -

• several times a week/sometimes -

• rarely/never -

6. When choosing a toothpaste or brush, what are you guided by? "The source of information"

• advice from a dentist -

• opinion of acquaintances or advertising -

• own opinion -

7. What kind of toothpaste does your child use when brushing their teeth?

• for children -

• for adults -

• another hygiene products – what? _______ Without toothpaste –

Made from natural ingredients –

8. When choosing a toothpaste, do you consider its composition?

• Yes -

• No -

9. How often do you change your child's toothbrush?

• once a year -

• once every 6 months -

• once every 3 months -

• once a month -

10. Does your child use an electric toothbrush?

• Yes -

• No -

11. From what sources did you get information about the Rules for brushing teeth?

• Internet/social networks -

• At the dentist's appointment –

• Acquaintances/relatives -

• TV and radio broadcast -

• I don't have any information –

12. Have you been examined and/or treated by a dentist?

• Yes -

• No -

13. For what reason did you visit the dentist?

• a) acute pain -

• b) routine check -

• c) dental problem (caries, plaque, etc.) -

• d) have not visited a dentist -

14. When was the last time you visited the dentist?

• _________________________________

15. Were there any problems with visiting the dentist?

• Yes -

• No -

If yes, what are the problems?

• ____________________________________________

Fear (of doctors, dental light, drill sounds, past negative extraction experience, etc.) –

Refuses to enter the dental office –

Refuses to sit in the dental chair –

Does not open mouth (does not understand the need to open it) –

Lacks sitting tolerance –

Long waiting time in public clinics –

Meltdown/tantrum –

16. Did you manage to carry out the treatment?

• Yes -

• No -

If "Yes" - how did you adapt to the dental treatment?

• _______________________________________________

Planning –

Under sedation –

General anesthesia –

With physical restraint –

17. Have you had general anesthesia or sedation during dental procedures?

• Yes -

• No-

18. In which clinic did you undergo dental treatment?

• state=

• private-

• did not pass-

19. How did you notice that the child has problems with his teeth?

• Grabbed his cheeks, put his fingers in his mouth

• Beat his mouth and cheeks

• Made sucking movements with lips

• Other_______________________________________________

20. Have there been cases when a child “sucked out” pieces of teeth?

•	Yes -

•	Not -

21.Where, by what specialists was Autism diagnosed?

• Center for mental health, child psychiatrist -

• Neurologist -

• Psychologist -

• Other -

• Not indicated anywhere -

22. At what age of a child did you notice peculiarities in his development?

___________________________________________

23. Were there any signs of “reverse development” (“rollback”)?

•	Yes

•	Not

If yes, at what age?

____________________________

24. Do you visit correctional centers?

•	Yes -

•	Not -

If “Yes”, please indicate which _____________________________

25. Do you think your child is a high functioning autist?

•	Yes -

•	Not -

26. Please briefly describe its features

Speech______________________________________________________________________

What stimmings (“stims”, motor stereotypes, repetitive behavior, self-stimulating behavior) ______________________________________________

What special habits _________________________________________________________

What communications methods _______________________________________________

27. What are the features of eating behavior?

Eats only one set of foods - list ____________________________

categorically refuses to eat meat products

For many years he eats only one food. Transfer________________________

28. Does your child eat sweets between meals?

•	Yes -

•	No -

• If using, how often? __________________

29. Does your child rinse his mouth after eating?

•	Yes -

•	No -

30. Does the child have atopic dermatitis?

•	Yes -

•	No -

31. What other diseases does the child have? ____________________________________________

32. Have you had a TMS (Tandem Mass Spectrometry) analysis for HBO (hereditary metabolic diseases)?

•	Yes -

•	Not -

If yes, what is the result? ____________________________________________

33. If there are tests for vitamins D3, Vit B12, Ferritin, hemoglobin, etc.

If “Yes”, please attach the most recent results of any blood, urine, stool tests

34. Do you have problems with constipation or frequent loose stools?

•	Yes -

•	Not -

35. Were you invited with your child to the polyclinic for psychophysical screening, to the CDR room (child development room) in the first years of life?

•	Yes -

•	No -

• Don't know about this -

36. Where does the child get an education?

• Inclusion -

• At home-

• In a mass team-

What are your suggestions for improving dental care for children with autism?
